# Macromineral requirements for maintenance and growth in male and female hair sheep

**DOI:** 10.3389/fvets.2023.1032429

**Published:** 2023-03-02

**Authors:** Caio J. L. Herbster, Matheus L. C. Abreu, Antonio S. Brito Neto, Marcilio S. Mendes, Luciano P. da Silva, Marcos I. Marcondes, Pedro H. S. Mazza, Luciano S. Cabral, Leilson R. Bezerra, Ronaldo L. Oliveira, Elzania S. Pereira

**Affiliations:** ^1^Department of Animal Science, Federal University of Ceara, Fortaleza, Brazil; ^2^Department of Animal Science, Federal University of Mato Grosso, Cuiaba, Brazil; ^3^Department of Animal Science, Washington State University, Pullman, WA, United States; ^4^Department of Veterinary Medicine and Animal Science, Federal University of Bahia, Salvador, Brazil; ^5^Center of Health and Agricultural Technology, Federal University of Campina Grande, Patos, Brazil

**Keywords:** minerals, models, retention, sex, sheep

## Abstract

A better understanding of the nutritional requirements of sheep, especially in terms of minerals, is crucial for improving production. We estimated the net requirements for Ca, P, K, Mg, and Na for gain (NCa_g_, NP_g_, NK_g_, NMg_g_, and NNa_g_) and maintenance (NCa_m_, NP_m_, NK_m_, NMg_m_, and NNa_m_) in male and female hair sheep. Six datasets with 248 individual records of hair sheep (139 non-castrated males, 75 castrated males and 34 females) were used to estimate the net macromineral requirements for gain. To estimate the net macromineral requirements for maintenance, 52 observations (26 non-castrated and 26 castrated males) were used. A meta-analytical approach was applied, using non-linear mixed effects models and the study as a random effect. Based on information criteria for model selection, heterogeneous variance functions were more likely to describe mineral requirements with a low level of model selection uncertainty. The adopted criteria allowed the choice of the best models to represent the macromineral requirements. The chosen models explained the observed variability in the sex, and the choices were based on a low level of uncertainty (w ≥ 0.90). Irrespective of sex, NCa_g_ and NP_g_ decreased with increasing BW from 10 to 30 kg and average daily gain (ADG) of 150 g/day, ranging from 1.71–1.38; 1.83–1.57; 1.82–1.51 of Ca and 0.86–0.66; 0.92–0.78; 0.92–0.75 of P for non-castrated males, castrated males, and females, respectively. The NK_g_ remained constant, with mean values of 0.26 g/day. The NNa_g_ range was 0.17 to 0.14 g/day for non-castrated males, 0.20 to 0.25 g/day for females, and constant (0.18 g/day) for castrated males with an increase in BW from 10 to 30 kg and an ADG of 150 g/day. Macromineral requirements for maintenance (mg/kg BW) and retention (%) were 23.70 and 54.30 for Ca, 25.33 and 79.80 for P, 11.74 and 5.00 for K, 2.63 and 8.50 for Mg, and 7.01 and 8.10 for Na for males. The International Committees did not provide inferences about the sex influence on mineral requirements. Our study indicates that sex is one factor that influences the macromineral requirements for gain. The information generated in this study can be used to optimize the mineral management of hair sheep in the growing phase in tropical regions.

## 1. Introduction

Hair sheep are still important animals in many regions of the world, especially in developing countries, where the demand for food products increases with population growth and associated needs. Although the FAOSTAT (1) has not numerically presented the growth rate of sheep in recent years, it reports that the population of small ruminants remains significant.

A better understanding of the nutritional requirements of sheep, especially in terms of minerals, is crucial for improving production. Minerals are essential elements and perform specific functions in the animal's body, accounting for 3.5% of the animal's body weight (2). Information on mineral requirements for growth (3, 4) and maintenance (5) are important to achieve the maximum productive potential of a herd (6). The net maintenance requirement represents the amount of mineral needed to replace endogenous losses (7), while the gain requirement represents the amount of mineral deposited in the animal's body, usually expressed in g or mg per kg of live weight gained or retained protein (8).

The mineral requirements of growing hair sheep is important, as it allows the reduction of costs in diet formulations in addition to reducing the environmental impact due to mineral contamination. Factors inherent to feed, bioavailability, and chemical form of the element, along with aspects related to inter-associations (antagonism and agonism) among minerals, breed or genetic group, and growth stage (9) can also influence dietary requirements.

Most nutritional requirement guidelines establish net mineral requirements for sheep during growing, however, variation in net mineral requirements across sexes has not been studied. Thus, there is a lack about sex effects on mineral requirements, and the International Committees (10–12) do not consider them in their recommendations. The sex is involved in animal physiology and metabolism and has effects on growth, body composition, and productive and reproductive functions. As with protein and energy, the sex modulates mineral dynamics and, in consequence, mineral deposition in the animal's body (13). Thus, the aim of this study was to estimate the net requirements for gain of Ca, P, K, Mg, Na (NCa_g_, NP_g_, NK_g_, NMg_g_, and NNa_g_) and maintenance (NCa_m_, NP_m_, NK_m_, NMg_m_, and NNa_m_) in male and female hair sheep. Thus, we hypothesized that the macromineral requirements of hair sheep differ between sexes.

## 2. Materials and methods

### 2.1. Database

To determine macromineral requirements for weight gain, the database consisted of six studies (5, 14–18), comprising a total of 248 animals, with 74 animals belonging to the reference group and 174 to the experimental groups and four breeds (Santa Ines, Morada Nova, Brazilian Somali and crossbreed) with three sex classes: non-castrated (*n* = 139), castrated (*n* = 75) males, and females (*n* = 34) (Table 1).

**Table 1:** Summary of studies used to estimation of net mineral requirements for the maintenance and gain of hair sheep.

**Study**	***n***	**Breed**	**Sex class**	**BW (kg)**	**EBW (kg)**
Cabral et al. (14)	24	Santa Ines	Castrated	17.90–24.40	13.13–22.65
Pereira et al. (15)	40	Morada Nova	Non-castrated	10.12–31.62	6.58–27.10
Silva et al. (16)	19	Crossbreed	Non-castrated	10.64–33.60	9.77–30.10
	17		Castrated	7.97–31.70	7.68–29.44
	18		Female	7.98–29.40	7.68–26.28
Pereira et al. (18)	47	Brazilian Somali	Non-castrated	10.90–35.74	7.95–31.18
Pereira et al. (5)^a^	14	Morada Nova	Non-castrated	13.66–38.28	9.62–29.38
	16		castrated	12.54–31.25	8.66–23.08
	16		Female	12.74–26.95	8.89–21.71
Pereira et al. (17)^a^	19	Santa Ines	Non-castrated	11.20–33.20	8.30–24.57
	18		Castrated	12.60–31.56	8.50–29.30

a Studies used in maintenance; BW, body weight; EBW, empty BW.

To estimate maintenance requirements, two studies were used (5, 17), comprising a total of 52 observations, with two sexes: non-castrated (*n* = 26) and castrated (*n* = 26) males. The descriptive analyses of the variables used in this study are provided in Tables 2, 3. For the meta-analytical approach, only studies containing information on individual animals fed at maintenance levels (12, 19) and at least two levels above maintenance were included. Furthermore, the studies were conducted with hair sheep raised in tropical regions and contained the following quantitative data on each animal: (i) body weight (BW); (ii) empty BW (EBW); and (iii) mineral body composition based on chemical analysis. All studies used the comparative slaughter method, in which the animals designated as the reference group were slaughtered at the beginning of the trial in order to estimate the initial body composition of those who remained in the study.

**Table 2:** Descriptive statistics of the data used to estimate the net requirements of macrominerals for gain in hair sheep.

**Item**	***n* ^a^**	**Mean**	**SD**	**Maximum**	**Minimum**
**Non-castrated males**					
BW (kg)	139	21.56	6.54	38.28	10.12
EBW (kg)	139	17.23	5.93	31.18	6.58
**Macromineral retention (g)**					
Ca	139	172.63	83.98	542.23	42.76
P	139	114.32	61.00	390.74	33.70
K	139	31.34	17.03	88.81	1.56
Mg	139	6.16	4.36	31.59	1.82
Na	139	25.22	14.93	77.29	2.02
**Castrated males**					
BW (kg)	75	21.50	5.76	31.70	7.97
EBW (kg)	75	16.81	4.87	29.44	7.68
**Macromineral retention (g)**					
Ca	75	235.45	75.02	395.48	89.33
P	75	133.13	53.59	268.26	55.49
K	75	33.14	19.08	69.74	2.42
Mg	51	6.16	4.36	31.59	1.82
Na	75	22.38	11.93	49.17	2.42
**Female**					
BW (kg)	34	18.91	5.36	29.40	7.98
EBW (kg)	34	16.03	4.99	26.28	7.68
**Macromineral retention (g)**					
Ca	34	216.90	85.68	547.40	92.96
P	34	144.56	64.36	394.93	66.57
K	34	20.85	19.16	60.80	2.64
Mg	34	6.97	6.27	34.99	2.34
Na	34	16.23	14.04	52.30	2.69

a *n*, number of records; BW, body weight; EBW, empty body weight; SD, standard deviation.

**Table 3:** Descriptive statistics of the data used to estimate the net requirements of macrominerals for maintenance in hair sheep.

**Item**	***n* ^a^**	**Mean**	**SD**	**Maximum**	**Minimum**
**Non-castrated males**					
BW (kg)	26	25.28	6.00	38.20	16.46
EBW (kg)	26	19.00	4.96	29.28	11.59
**Macromineral intake (mg/day)**					
Ca	26	142.28	41.73	220.01	77.31
P	26	90.22	23.92	140.69	59.67
K	26	441.27	93.75	575.28	278.37
Mg	26	63.92	16.65	94.78	37.55
Na	26	169.60	38.05	225.04	105.72
**Macromineral retention (mg/day)**					
Ca	26	62.40	33.06	124.92	10.31
P	26	47.45	20.58	87.97	11.76
K	26	11.39	5.33	19.43	0.09
Mg	26	2.50	1.91	7.27	0.04
Na	26	6.85	4.03	13.35	−0.17
**Castrated males**					
BW	26	23.09	5.13	31.56	15.98
EBW	26	17.47	4.38	25.15	11.37
**Macromineral intake (mg/day)**					
Ca	26	143.53	44.89	232.47	77.44
P	26	89.46	25.14	149.78	59.05
K	26	441.32	100.29	622.32	278.84
Mg	26	63.24	16.35	92.99	37.62
Na	26	171.68	43.68	255.66	105.91
**Macromineral retention (mg/day)**					
Ca	26	48.72	27.00	98.05	2.99
P	26	36.06	22.19	72.05	2.87
K	26	9.39	5.16	18.16	0.97
Mg	26	3.08	2.50	8.90	−0.31
Na	26	6.46	3.65	12.78	1.02

a *n*, number of records; BW, body weight; EBW, empty body weight; SD, standard deviation.

### 2.2. Chemical analyses

The EBW and mineral composition of reference animals (Supplementary Table S1) were used to estimate the initial EBW, and the mineral body composition of the remaining animals slaughtered at the end of the trials, within each trial. The macromineral retention was calculated as the final mineral content minus the initial mineral content estimated from the reference animals.

The body components (i.e., blood, hide, hooves, head, organs, and right half of the carcass) were homogenized, sampled, and placed in a forced ventilation oven at 55°C for 72 h. Posteriorly, these samples were defatted by extraction in a Soxhlet apparatus for 12 h [(20); method 920.39]. Subsequently, the fat-free body components samples were ground in a ball mill and analyzed for DM [(20); method 967.03], ash [(20); method 942.05], and CP [(20); method 981.10] levels. The body water content was determined as 100% minus the body component DM. Samples of rations, feed, orts, and body components of each study were analyzed for mineral composition through digestion in nitroperchloric acid, according to method INCT-CA M-004/1 (21). The Ca and Mg were determined by adding strontium chloride and read using atomic absorption spectrometry [GBC Avanta Sigma, Hampshire, USA; (22); method 968.08]. The P was determined by reducing the phosphorus-molybdate complex with ascorbic acid, followed by reading with a colorimetric spectrophotometer [(22); method 965.17]. The Na and K concentrations were determined by flame emission spectrometry [Corning 400, NY, USA; (22); method 985.35]. Further details about slaughter procedures, experimental diets, and sample collection of body components can be found in each study (5, 14–18).

### 2.3. Models and calculations

#### 2.3.1. Net macromineral requirements for gain

To estimate the requirements for gain, initially, the body mineral composition in the EBW was estimated by using the Brody (23) model, as shown in Equation 1:


(1) $$\begin{array}{c} RM=\;\beta 0\;\times \;EBW^{\beta 1} \end{array}$$

where RM = retained mineral (mg/kg EBW/day); EBW = empty BW (kg); β0 and β1 are parameters of the equation. Thus, the net macromineral requirements for gain were estimated by first derivation of Equation 1, as follows in Equation 2:


(2) $$\begin{array}{c} NRg=EBWG\;\times \;\left(\beta 0\;\times \;\beta 1\;\times \;EBW^{\left(\beta 1-1\right)}\right) \end{array}$$

where NRg = net mineral requirements for weight gain (g/day); EBW = empty BW (kg); EBWG = empty BW gain (kg); β0 and β1 are parameters of the equation.

#### 2.3.2. Net macromineral requirements for maintenance

A linear regression generated from the relationship between retained minerals (mg/kg BW) against mineral intake (mg/kg BW) was used to calculate the net macromineral requirements for maintenance (mg/kg BW), according to Equation 3:


(3) $$\begin{array}{c} RM=\;\beta 0+\;\beta 1\;\times \;MI \end{array}$$

where, RM = retained mineral (mg/kg BW/day); MI = mineral intake (mg/kg BW/day); β0 and β1 are regression parameters, β0 is considered the net requirement of each mineral for maintenance (mg/kg BW/day), and β1 is the slope, considered the retention coefficient (%). However, only studies of Pereira et al. (5, 17) were considered to estimate maintenance requirements (Table 2). The estimated requirements based on the EBW were converted to the BW using the factor (1.23) derived from the relationship between BW/EBW (24). The dietary requirements were calculated as a sum of the net requirements for maintenance and gain divided by the retention coefficient.

### 2.4. Statistical analysis

#### 2.4.1. Model adjustments for gain

The allometric model was applied to predict the retention (mg/kg EBW/day) of macrominerals in relation to EBW (kg) (Equation 4):


(4) $$\begin{array}{c} RM_{Y}=\alpha X^{\beta } \end{array}$$

(5) $$\begin{array}{c} RM_{Y_{i}}=\alpha_{i}{X_{i}}^{\beta_{i}} \end{array}$$

The model presented in Equation 5 differs from Equation 4 because the prediction of mean body retention of the Y-th (Y = 1, 2…, 5) mineral (RM_Yi_) was separated by the i-th non-castrated males, castrated males, and females, that is, i = 1, 2, and 3. Similarly, the estimates of the parameters αi and βi represent estimated values separated by i-sex.

#### 2.4.2. Model adjustments for maintenance

The linear model, Equation 6, was applied for predictions of the macromineral requirements for maintenance in non-castrated and castrated males.


(6) $$\begin{array}{c} RM_{Y}=\alpha +\beta X \end{array}$$

The unit of measurement of RMy was according to the Y-th (Y = 1, 2…, 5) mineral. The parameters β_0_ and β_1_ are the intercept and slope, respectively.

#### 2.4.3. Description of variance functions and combinations

The variance ($\sigma_{Y}^{2}$) was also modeled to test its homoscedasticity assumption. In this case, three variance functions were tested, one homogeneous (homoscedastic) and two heterogeneous functions:


(7) $$\begin{array}{c} \sigma_{Y}^{2}=\sigma^{2} \end{array}$$

(8) $$\begin{array}{c} \sigma_{Y}^{2}=\sigma^{2}\exp\left(\rho X\right) \end{array}$$

(9) $$\begin{array}{c} \sigma_{Y}^{2}=\sigma^{2}|RM_{Y}{|}^{2\phi } \end{array}$$

(10) $$\begin{array}{c} \sigma_{Y_{i}}^{2}=\sigma_{i}^{2} \end{array}$$

(11) $$\begin{array}{c} \sigma_{Y_{i}}^{2}=\sigma_{i}^{2}\exp\left(\rho X_{i}\right) \end{array}$$

(12) $$\begin{array}{c} \sigma_{Y_{i}}^{2}=\sigma_{i}^{2}|RM_{Y_{i}}{|}^{2\phi } \end{array}$$

(13) $$\begin{array}{c} \sigma_{Y_{i}}^{2}=\sigma^{2}\exp\left(\rho_{i}X_{i}\right) \end{array}$$

(14) $$\begin{array}{c} \sigma_{Y_{i}}^{2}=\sigma^{2}|RM_{Y_{i}}{|}^{2\phi_{i}} \end{array}$$

(15) $$\begin{array}{c} \sigma_{Y_{i}}^{2}=\sigma_{i}^{2}\exp\left(\rho_{i}X_{i}\right) \end{array}$$

(16) $$\begin{array}{c} \sigma_{Y_{i}}^{2}=\sigma_{i}^{2}|RM_{Y_{i}}{|}^{2\phi_{i}} \end{array}$$

The parameter σ refers to the standard deviation, and the term σ^2^ refers to the random error variance; thus, Equation 7 represents the homogeneous variance with the assumption of homoscedasticity (25, 26). In this case, the homogeneous (Equations 7 and 10), exponential (Equations 8, 11, 13, and 15), and Power-of-the-Means (Equations 9, 12, 14, and 16) variance functions were tested, as they are the most commonly used (27–29). An attempt to accommodate heteroscedasticity was performed with Equations 8 to 16. Due to the possible scaling effect on the variability, two functions (Equations 8 and 9) were used to adjust for the possible scaling effect on the variance (26). The exponential function represented by Equation 8 assumes an exponential increase in the variance σ^2^ with an increasing rate (“ρ”) as a function of the EBW, represented by the term X. Equation 9 is the Power-of-the-Means Function for the absolute value of RM_Y_, with a scaling parameter φ. Both the parameters ρ and φ are dimensionless, with values ranging from –∞ to ∞.

For Equations 7–9, a single variance ($\sigma_{Y}^{2}$) was estimated independent of the i-th sex. Equations 8–16 use heterogeneous variance functions as they describe a non-linear behavior, except for Equation 10. In Equation 10, the heterogeneous behavior was attributed to the $\sigma_{i}^{2}$ being estimated for the i-th sex with homogeneous variance. Regarding Equations 11, 12, the adjustments occurred with a distinct σ^2^ for each i-sex, whereas the parameters ρ and φ were common for the database. Contrary to the previous equations, in Equations 13 and 14, the individualized variance function parameters by sex were ρ_i_ and φ_i_, with σ^2^ being common. The most complex variance functions are described in Equations 15 and 16 as all their parameters are individualized by sex; $\sigma_{i}^{2}$, ρ_i_ (Equation 15), and φ_i_ (Equation 16).

As Equation 4 was combined with the variance functions (Equations 7–9), three models were fitted to the entire dataset of a given mineral, with no sex effect. On the other hand, Equation 5 was combined with Equations 7–16, totaling 10 different combinations. Initially, adding the combinations made with Equations 4 and 5, 13 combinations were made. Subsequently, all these 13 combinations were performed again, but we added the effect of studies on the parameters “α” and “β” (Equation 4) and α_i_ and β_i_ (Equation 5). Finally, 26 combinations were performed for the body retention of each analyzed mineral. The linear model, Equation 6, was combined with the variance functions demonstrated in Equations 7–9, with three models being fitted to each mineral, with no sex effect.

The fits of the model combinations, both retention and maintenance were performed by the procedure for mixed non-linear models – NLMIXED of SAS. The algorithm used to estimate the maximum likelihood method was the Newton–Raphson (tech = NEWRAP). The assumption of normality was assumed and therefore Y, and the covariance between the measurements was considered null since these were not taken in the same experimental unit. The corrected Akaike information criterion (AICc), the variation (Δ) between each model used and the model with the smallest AICc, the model probability (w), and the evidence ratio (ER) were calculated for model evaluation. Twelve predictions of mineral retention were performed between 8 and 30 kg of EBW (Equation 17) by the derivative of Equation 4 (29):


(17) $$\begin{array}{c} \frac{dRM_{Y}}{dX}=\hat{\alpha \beta X^{\beta -1}} \end{array}$$

The mean predictions of retention rates and their respective 95% confidence intervals (95% CI; Equation 18) were estimated from the best fits for each mineral evaluated. The 95% confidence intervals (95% CI; Equation 18) were also estimated for the parameters “α” and “β” (Equation 4) and α_i_ and β_i_ (Equation 5):


(18) $$\begin{array}{c} 95\%\;CI=mean\pm \;t_{\left(1-\propto ,\;df\right)}SE \end{array}$$

The term mean refers to the average prediction of the model as well as the parameters. The expression ± *t*_(1−∝, *df*)_ SE represents the estimate of the critical value of the two-tailed distribution of the Student's *t-*test using the significance level α = 0.05 and degree of freedom (df = n–θ), where n represents the number of observations and θ corresponds to the number of parameters of the allometric model (Equations 4 and 5) as well as the variance functions represented in Equations 7–16.

## 3. Results

Combining Equations 4 and 5 with the variance functions described by Equations 7–16 enabled the estimation of the requirements for gain of NCa_g_, NP_g_, NMg_g_, and NNa_g_ for castrated males, non-castrated males and females. A general equation was generated for NK_g_ (Table 4). The adopted criteria allowed the choice of the best models to represent the requirements for gain, explaining the observed variability among sexes (Table 5).

**Table 4:** Models for estimating body composition and net requirements for gain of hair sheep.

**Macrominerals**	**Sex**	**Model**	**Variance parameters**	**Net requirements for gain^a^ (g/day)**
Ca	Non-castrated males	20.41 (4.31) EBW^0.83 (0.10)^	*σ =* 21.14; *ρ =* 0.06	EBWG [(17.03) EBW^−0.16^]
	Castrated males	19.02 (4.29) EBW^0.88 (0.10)^	*σ =* 21.14; *ρ =* 0.05	EBWG [(16.75) EBW^−0.12^]
	Female	20.53 (4.36) EBW^0.85 (0.11)^	*σ =* 21.14; *ρ =* 0.09	EBWG [(17.52) EBW^−0.14^]
P	Non-castrated males	11.57 (1.31) EBW^0.79 (0.06)^	*σ =* 4.69; *ρ =* 0.14	EBWG [(9.19) EBW^−0.20^]
	Castrated males	9.96 (1.40) EBW^0.86 (0.07)^	*σ =* 4.69; *ρ =* 0.12	EBWG [(8.65) EBW^−0.13^]
	Female	10.94 (1.55) EBW^0.83 (0.08)^	*σ =* 4.69; *ρ =* 0.22	EBWG [(9.16) EBW^−0.16^]
K	All sex	1.98 (0.52) EBW^0.99 (0.10)^	*σ =* 1.07; *φ =* 0.47	EBWG [(1.96) EBW^−0.01^]
Mg	Non-castrated males	0.49 (0.08) EBW^0.91 (0.12)^	*σ =* 0.24; *φ =* 0.91	EBWG [(0.45) EBW^−0.08^]
	Castrated males	0.37 (0.08) EBW^1.04 (0.12)^	*σ =* 0.12; *φ =* 1.18	EBWG [(0.39) EBW ^0.04^]
	Female	0.48 (0.09) EBW^0.935 (0.13)^	*σ =* 0.05; *φ =* 1.88	EBWG [(0.45) EBW^−0.06^]
Na	Non-castrated males	1.99 (0.34) EBW^0.83 (0.09)^	*σ =* 1.05; *φ =* 0.52	EBWG [(1.67) EBW^−0.16^]
	Castrated males	1.19 (0.18) EBW^1.031 (0.08)^	*σ =* 1.05; *φ =* 0.23	EBWG [(1.23) EBW^0.03^]
	Female	0.87 (0.23) EBW^1.18 (0.10)^	*σ =* 1.05; *φ =* 0.246	EBWG [(1.03) EBW^0.18^]

a The empty body weight gain (EBWG g/day) was calculated according to Herbster et al. (24); EBW, empty body weight.

**Table 5:** Information criteria of fitted models to estimate the net requirements for gain of hair sheep.

**Macrominerals**	**Model**	**Variance functions**	**Random effect**	**AICc**	**Δ**	**w**	**ER**	**Θ**
Ca	Equation (5)	Equation (12)	α_i_; β_i_	2563.90	0.00	0.32	1.00	16.00
	Equation (5)	Equation (15)	α_i_; β_i_	2564.40	0.50	0.25	1.28	18.00
	Equation (5)	Equation (10)	α_i_; β_i_	2564.80	0.90	0.20	1.57	16.00
	Equation (5)	Equation (7)	α_i_; β_i_	2566.60	2.70	0.08	3.86	14.00
	Equation (4)	Equation (7)	α; β	2566.90	3.00	0.07	4.48	6.00
P K	Equation (5)	Equation (12)	α_i_; β_i_	2167.40	0.00	1.00	1.00	16.00
	Equation (4)	Equation (8)	α; β	1551.10	0.00	0.92	1.00	6.00
Mg	Equation (5)	Equation (11)	α_i_; β_i_	779.70	0.00	0.98	1.00	16.00
Na	Equation (5)	Equation (13)	α_i_; β_i_	1391.30	0.00	0.80	1.00	16.00
	Equation (5)	Equation (11)	α_i_; β_i_	1394.20	2.90	0.19	4.26	16.00

AICc, Akaike information criterion corrected for small samples; Δ, Akaike difference; w, model probability; ER, evidence ratio computed for each model; Θ, number of parameters of the allometric model.

Irrespective of sex, NCa_g_ and NP_g_ decreased with increasing BW from 10 to 30 kg and average daily gain (ADG) of 150 g/day, ranging from 1.71–1.38; 1.83–1.57; 1.82–1.51 of Ca and 0.86–0.66; 0.92–0.78; 0.92–0.75 of P for non-castrated males, castrated males, and females, respectively. The NMg_g_ independent of BW and gain remained constant for each sex. Females during growth presented greater requirements than non-castrated males for Ca, P, Mg and Na. The NK_g_ remained constant, with mean values of 0.26 g/day. The NNa_g_ range was 0.17–0.14 g/day for non-castrated males, 0.20–0.25 g/day for females, and constant (0.18 g/day) for castrated males with an increase in BW from 10 to 30 kg and an ADG of 150 g/day (Table 6).

**Table 6:** Net mineral requirements for gain of hair sheep with respective confidence intervals.

**BW (kg)** **ADG (g/day)**	**10**			**20**			**30**		
	**100**	**150**	**200**	**100**	**150**	**200**	**100**	**150**	**200**
**Non-castrated males**									
Ca	1.14 ± 0.64	1.71 ± 0.96	2.28 ± 1.28	0.99 ± 0.77	1.49 ± 1.15	1.99 ± 1.54	0.92 ± 0.83	1.38 ± 1.24	1.84 ± 1.66
P	0.57 ± 0.24	0.86 ± 0.36	1.14 ± 0.48	0.48 ± 0.27	0.72 ± 0.40	0.96 ± 0.54	0.44 ± 0.28	0.66 ± 0.42	0.88 ± 0.56
Mg	0.03 ± 0.03	0.05 ± 0.05	0.07 ± 0.07	0.03 ± 0.04	0.05 ± 0.06	0.07 ± 0.08	0.03 ± 0.04	0.05 ± 0.07	0.06 ± 0.09
Na	0.11 ± 0.09	0.17 ± 0.13	0.22 ± 0.18	0.10 ± 0.09	0.15 ± 0.14	0.20 ± 0.19	0.09 ± 0.10	0.14 ± 0.15	0.18 ± 0.20
**Castrated males**									
Ca	1.22 ± 0.65	1.83 ± 0.98	2.44 ± 1.30	1.10 ± 0.82	1.66 ± 1.23	2.21 ± 1.64	1.05 ± 0.90	1.57 ± 1.36	2.09 ± 1.81
P	0.62 ± 0.24	0.92 ± 0.36	1.23 ± 0.48	0.55 ± 0.29	0.83 ± 0.44	1.10 ± 0.59	0.52 ± 0.32	0.78 ± 0.48	1.04 ± 0.64
Mg	0.04 ± 0.03	0.06 ± 0.05	0.08 ± 0.07	0.04 ± 0.05	0.06 ± 0.07	0.08 ± 0.09	0.04 ± 0.05	0.06 ± 0.08	0.08 ± 0.11
Na	0.12 ± 0.10	0.18 ± 0.16	0.24 ± 0.21	0.12 ± 0.12	0.18 ± 0.19	0.24 ± 0.25	0.12 ± 0.14	0.18 ± 0.21	0.25 ± 0.28
**Females**									
Ca	1.21 ± 0.72	1.82 ± 1.08	2.43 ± 1.45	1.07 ± 0.87	1.61 ± 1.31	2.15 ± 1.75	1.00 ± 0.95	1.51 ± 1.42	2.01 ± 1.90
P	0.62 ± 0.29	0.92 ± 0.44	1.23 ± 0.59	0.54 ± 0.35	0.81 ± 0.52	1.07 ± 0.69	0.50 ± 0.37	0.75 ± 0.56	1.00 ± 0.74
Mg	0.05 ± 0.03	0.07 ± 0.05	0.09 ± 0.07	0.05 ± 0.04	0.07 ± 0.06	0.09 ± 0.09	0.05 ± 0.05	0.07 ± 0.07	0.09 ± 0.10
Na	0.13 ± 0.14	0.20 ± 0.21	0.26 ± 0.27	0.15 ± 0.18	0.23 ± 0.27	0.31 ± 0.37	0.17 ± 0.21	0.25 ± 0.32	0.33 ± 0.43
**All sex**									
K	0.17 ± 0.15	0.26 ± 0.23	0.35 ± 0.31	0.17 ± 0.18	0.26 ± 0.27	0.35 ± 0.36	0.17 ± 0.20	0.26 ± 0.30	0.34 ± 0.40

BW, body weight; ADG, average daily gain.

**Figure 1:**
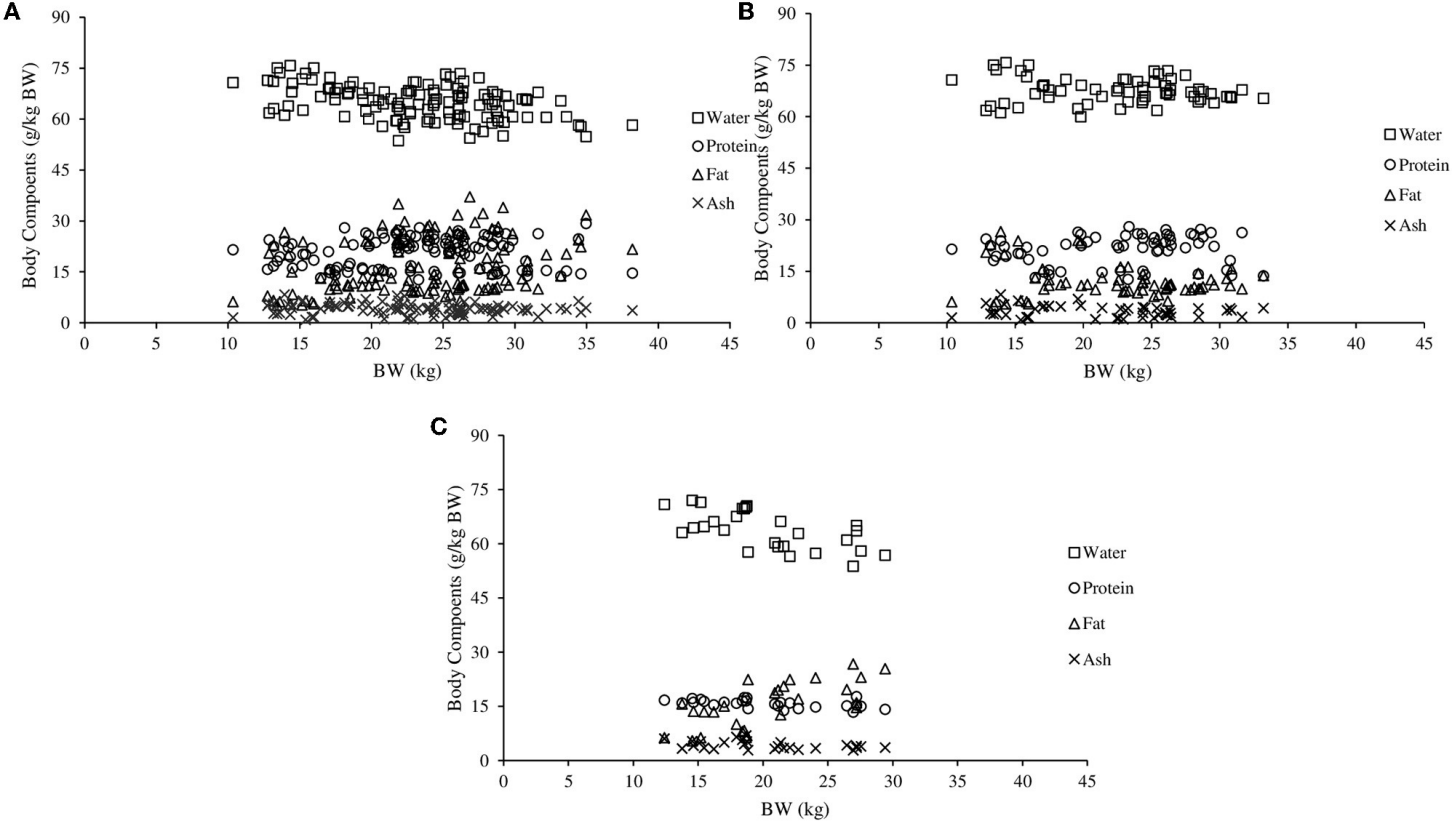
Proportions of water, protein, fat and ash in non-castrated males **(A)**, castrated males **(B)**, and females **(C)** hair sheep.

The Figure 1 shows the amount of water, protein and ash in relation to BW increased from 10 to 30 kg. In the respective weight range, when the 95% confidence limits of NCa_g_, NP_g_, NMg_g_, NNa_g_, and NK_g_ were evaluated, uncertainty irrespective of sex increased as BW increased. However, when the NMg_g_ was evaluated, uncertainty for females was intermediary compared to non-castrated and castrated males.

**Figure 2:**
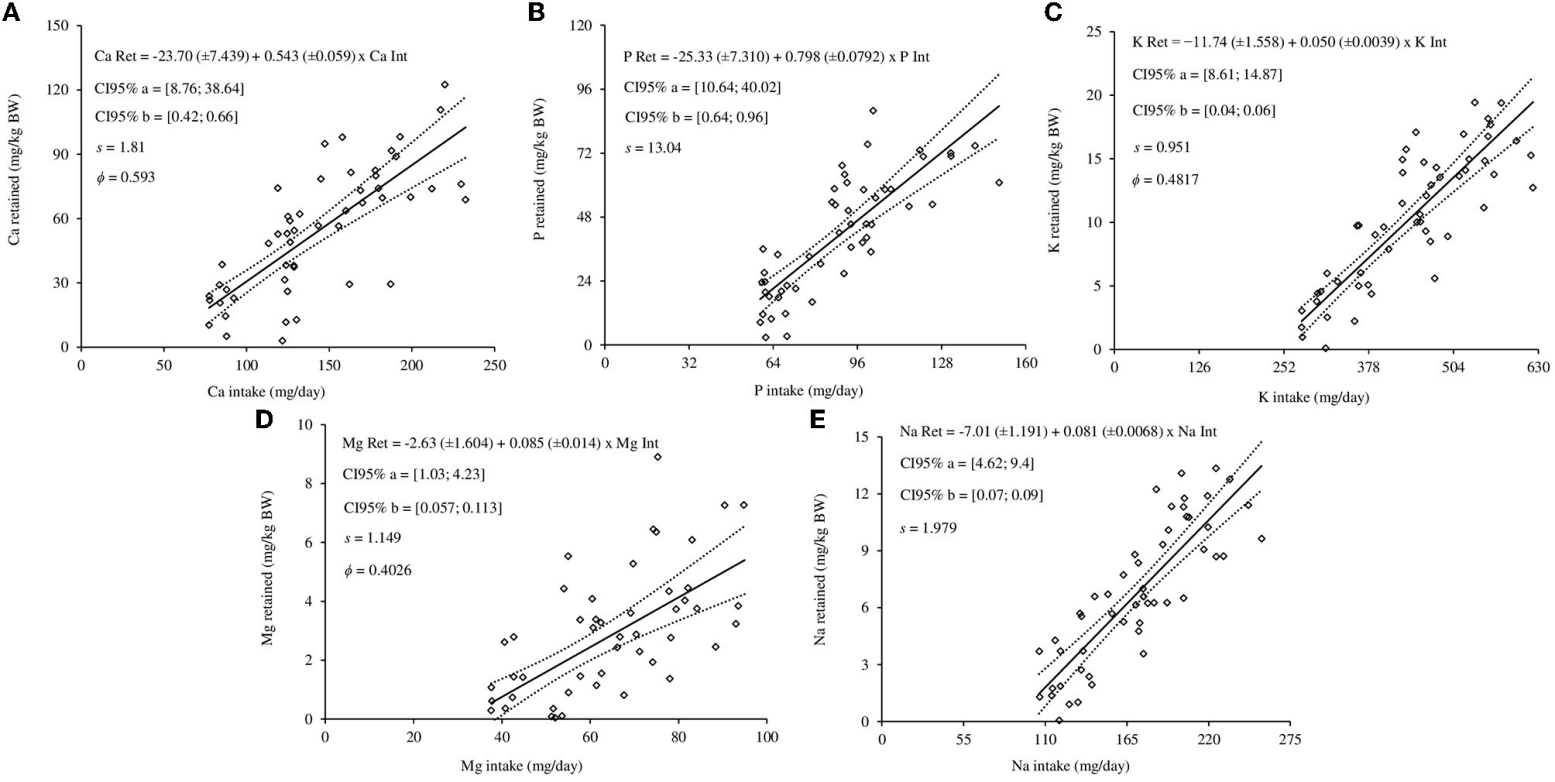
Regression equations of the relationships between retentions (Ret) and intakes (Int) of calcium **(A)**, phosphorus **(B)**, potassium **(C)**, magnesium **(D)**, and sodium **(E)** of hair sheep. The diamonds are the observed values of macromineral retention for maintenance; the solid line represents estimated values; dashed lines represent lower and upper values of the 95% confidence interval (95% CI); *a* and *b* are the equation parameters; s is the standard deviation; ϕ is the scale parameter.

Macromineral requirements for maintenance were estimated by the relationships between minerals retained and mineral intake, combing the linear model (Equation 6) with the variance functions described by Equations 7–9. Sex did not affect the intercept and slope of the linear equations to estimate the Ca (Figure 2A), P (Figure 2B), K (Figure 2C), Mg (Figure 2D), and Na (Figure 2E), therefore, linear equations were generated for each macromineral. The best model to represent the mineral requirements for maintenance was based on the adopted criteria shown in Table 7.

**Table 7:** Information criteria of fitted models to estimate the net requirements for maintenance of hair sheep.

**Macrominerals**	**Variance functions**	**AICc**	**Δ**	**w**	**ER**	**Θ**
Ca	Equation (4)	452.20	2.90	0.14	4.26	3.00
	Equation (5)	451.20	1.90	0.24	2.59	4.00
	Equation (6)	449.30	0.00	0.62	1.00	4.00
P	Equation (4)	405.20	0.00	0.44	1.00	3.00
	Equation (5)	406.30	1.10	0.25	1.73	4.00
	Equation (6)	405.90	0.70	0.31	1.42	4.00
K	Equation (4)	263.50	2.20	0.19	3.00	3.00
	Equation (5)	263.10	1.80	0.23	2.46	4.00
	Equation (6)	261.30	0.00	0.57	1.00	4.00
Mg	Equation (4)	205.10	1.80	0.22	2.46	3.00
	Equation (5)	205.00	1.70	0.23	2.34	4.00
	Equation (6)	203.30	0.00	0.55	1.00	4.00
Na	Equation (4)	170.80	0.00	0.62	1.00	3.00
	Equation (5)	173.20	2.40	0.19	3.32	4.00
	Equation (6)	173.20	2.40	0.19	3.32	4.00

AICc, Akaike information criterion corrected for small samples; Δ, Akaike difference; w, model probability; ER, evidence ratio computed for each model; Θ, number of parameters of the linear model.

The net mineral requirement for maintenance and retention were 23.70 mg/kg and 54.30% for Ca, 25.33 mg/kg and 79.80% for P, 11.74 mg/kg and 5.00% for K, 2.63 mg/kg and 8.50% for Mg, 7.01 mg/kg BW and 8.1% for Na, respectively (Figure 2). When the 95% confidence limits of requirements for maintenance of Ca and P were evaluated, the uncertainty of maintenance for males was greater. In addition, the uncertainty of Mg, Na, and K for maintenance were lower. The dietary mineral requirements are shown in Table 8.

**Table 8:** Dietary mineral requirements for hair sheep.

**BW** **(kg)**	**ADG (g/day)**	**Non-castrated males**				**Castrated males**				**Female**				**All sex**
		**Dietary minerals requirements (g/day)**												
		**Ca**	**P**	**Mg**	**Na**	**Ca**	**P**	**Mg**	**Na**	**Ca**	**P**	**Mg**	**Na**	**K**
10	100	2.54	1.03	0.77	2.25	2.68	1.09	0.81	2.32	2.67	1.09	0.92	2.48	5.83
	150	3.59	1.39	0.98	2.95	3.81	1.48	1.05	3.05	3.79	1.48	1.20	3.29	7.58
	200	4.64	1.75	1.20	3.64	4.93	1.86	1.29	3.78	4.91	1.86	1.47	4.09	9.32
20	100	2.70	1.24	1.06	2.94	2.91	1.33	1.16	3.23	2.85	1.31	1.25	3.61	8.15
	150	3.61	1.54	1.27	3.55	3.92	1.67	1.41	3.97	3.84	1.65	1.53	4.56	9.88
	200	4.53	1.84	1.47	4.15	4.94	2.02	1.66	4.72	4.83	1.98	1.80	5.50	11.61
30	100	3.01	1.50	1.38	3.72	3.24	1.60	1.50	4.11	3.16	1.58	1.58	4.64	10.48
	150	3.85	1.78	1.57	4.29	4.20	1.93	1.76	4.87	4.09	1.89	1.86	5.66	12.20
	200	4.70	2.05	1.77	4.85	5.16	2.25	2.01	5.63	5.01	2.20	2.12	6.69	13.92

BW, body weight; ADG, average daily gain.

## 4. Discussion

Information on mineral requirements for growth and maintenance is essential for herds to reach their maximum productive potential. The results of the present study emphasize the importance of updating the mineral recommendations for sheep considering the effect of sex.

As the animal grows, the body mineral content increases at similar rates early in life, decelerating later, and reaching a plateau after maturity (30, 31). One of the factors that affect this pattern of deposition mineral is sex class. Females have greater Ca and P requirements than non-castrated males. Sex may affect the mineral requirements for weight gain because females closer to maturity have higher levels of estrogen (32), which can modulate mineral dynamics in the body.

Braithwaite and Riazuddin (33) reported the effect of growth hormone on Ca metabolism, with a greater retention and an increased rate of bone Ca in ewes close to maturity. In this phase, Ca and P are mainly involved in the completion of bone development. Estradiol indirectly modulates bone Ca deposition, promoting the closure of the epiphyseal cartilage (34) due to the replacement of chondrocytes by osteogenic cells (35). Estrogen promotes osteoclast apoptosis (36) and thus decreases bone resorption, which explains why sex affects the requirements for Ca and P, implying greater requirements for these minerals for females.

The influence of sex in the modulation of the dynamics of nutrients and the metabolic activity of the tissues due to the variation in the levels of androgenic hormones (37) can also explain the uncertainty. Irrespective of sex, the uncertainty increased as BW increased from 10 to 30 kg of BW when the 95% confidence limits were evaluated. For Ca and P, this may be associated with the dilution effect caused by the increase in body fat, since fat has a low mineral content. Body fat is the item that varies the most, while fat-free dry mass is quite constant (31) since the main change in body composition that occurs with animal growth and development is the increase in fat content and this mechanism represents the degree of maturity of the animal.

This variation of body composition explains the higher levels of uncertainty with increasing BW. In our study, sheep weighing 30 kg and 150 g/day ADG had NCag and NPg values of 1.38, 1.57, and 1.51 g/day and 0.66, 0.78, and 0.75 g/day for non-castrated males, castrated males, and females, respectively. Regardless of sex class, the estimates of NCag and NPg were similar to those suggested by CSIRO (38), of 1.10 and 0.66 g/day, respectively. However, the CSIRO (38) considers weight at maturity in estimating gain, in addition to using different methodologies and genotypes from the present study.

In our study, the NCa_m_ and the retention coefficient were 23.70 mg/kg BW and 54.30%, respectively. Irrespective sex, the NCa_m_ for animals with 30 kg BW was 710 mg Ca/day (0.710g), corresponding to a dietary requirement of 4.05 g/day considering a Ca retention coefficient of 54.30%. This dietary Ca requirement was 29% higher than that recommended by the NRC (12) but 38% lower than that recommended by the INRA (39). The recommended NP_m_ in our study was 25.33 mg/kg BW and the retention coefficient was 80%. Irrespective of sex, hair sheep had a P dietary requirement of 1.87 g/day. This value is close to the NRC value (12), which is 1.98 g/day for sheep.

The estimation of macromineral requirements for sheep varies between the different Committees, being the main factors for variation, the differences in the values adopted for maintenance requirements and the coefficient of absorption or retention of minerals. The contrasting results of these coefficients are due to endogenous losses that are associated with variations in voluntary intake that directly influence maintenance requirements.

The use of the true retention coefficient seems to be more accurate than the absorption coefficient when it comes to estimating mineral requirements, as the minerals being absorbed will not always be used by the animal. It is important to emphasize that mineral requirements must be considered, as the exact demand for mineral needs can reduce mineral excretion and environmental pollution, especially that caused by excessive phosphorus excretion. The imbalance in P levels contributes to soil contamination, which when carried by rain can contribute to water eutrophication (40). The proposal of the equations for P aims to meet the requirements of growing animals, minimizing the use of P in the diet because high dietary concentrations may increase fecal and urinary excretion of the mineral.

Differences in the requirements for Mg were observed for animals during the evaluated growth phase. The relationship between Mg and energy metabolism may explain the effects of sex on the requirements of female hair sheep. Commonly, females initiate fat deposition more quickly when compared to males (12). Thus, the composition of the gain could explain the higher Mg requirement in females, compared to males, as they deposit greater amounts of fat (41).

Considering the role of Mg in the cell's energy metabolism, it is likely that the Mg requirements of females are in line with the greater energy requirements for maintaining females at the peak of growth, coinciding with sexual maturity. In addition, in the closing of the bone epiphysis, a greater supply of vitamin D is required because various stages of vitamin D conversion are actively dependent on the bioavailability of Mg (42, 43), contributing to maintaining the physiologic functions of the musculoskeletal system.

Magnesium, along with K and Na, is a cation extensively distributed in the body, around 70% is associated with the skeleton, 25% with the muscle mass, and only around 1% within the extracellular space (44). Since Mg metabolism is not considered to be under hormonal control, the variation of body composition and, consequently of Mg in the bone and tissues can explain the uncertainty as the animal grows and approaches maturity.

In the current study, sex did not affect K and Na requirements for maintenance. The Na and K are required for nutrient absorption processes in the gastrointestinal tract, and for maintenance of the acid-base balance in the animal's body (9). All these processes are essential for life. Thus, due to the role in homeostatic control, the requirement for maintenance did not vary between sex. In our study, the requirements for K and Na were 0.35 and 0.21 g/day, respectively, for the maintenance of hair sheep with 30 kg of BW. These values are lower than those recommended by the NRC (12) and INRA (39) that suggests 4.10 and 3.15 g/day for K and 0.32 and 0.45 g/day for Na, respectively. Thus, our data indicate that both Committees overestimate K and Na requirements for the maintenance of hair sheep.

Sex did not affect K requirements for growth. For animals weighing 30 kg BW with 150 g ADG (136 g EBWG), the NK_g_ correspond to 0.258 g/day. In this context, the value suggested by our study is close to the recommendations of the NRC (12) and INRA (39), which corresponds to 0.270 g/day. However, the sex influences NNa_g_, and females demand greater amounts of Na with increasing BW compared to males. Regarding the ARC (7) and Suttle (9), the Na recommendation for sheep is around 1.10 g/kg of weight gain, a value also suggested by the NRC (12). The body concentration of Na is about 1.3 g/kg BW (38), of which about a third is in the bones and most of the remainder in the extracellular fluids (2). Sodium present in bones is adsorbed on the surfaces of hydroxyapatite crystals within long bones and therefore does not interact with other ions present in body fluids (45).

The Na requirements are related to metabolic changes that occur as the animal grows. There is an inverse relationship between water and fat in the animal's body; with increasing fat content, the water content decreases (3). The increase in body fat deposition in females close to mature weight can alter the body Na balance. This occurs because the reduction in water content increases the concentration of Na in the extracellular fluid, which increases the glomerular filtration rate and reduces the efficiency of tubular absorption. These metabolic changes increase the Na excretion rates and may therefore be associated with a higher Na requirement as the BW increases (45).

In conclusion, our study brings a significant contribution since it shows the influence of sex on mineral requirements of hair sheep breed of Santa Ines, Morada Nova, Brazilian Somali and crossbreed. The confidence limits show that the models proposed to estimate the nutritional requirements of macrominerals for growing hair sheep were viable. This represents an advance in the understanding of nutritional requirements because the confidence limits in mineral requirements has not been reported by the committees, and requirements are considered as mean values. The recommendations for confined sheep of different sexes in the growth phase of 10 to 30 kg BW allow the nutritionist to recommend the mineral requirements more reliably, which implies a reduction in costs and environmental impact. The information generated in this study can be used to optimize the mineral management of hair sheep in the growing phase in warm areas.

## Data availability statement

The original contributions presented in the study are included in the article/Supplementary material, further inquiries can be directed to the corresponding author.

## Ethics statement

The approval of the ethics committee on animal use was not necessary for this study because the data were collected from previously published sources.

## Author contributions

CH, AB, and MMe: data curation and investigation. MA, LS, and MMa: methodology, formal analysis, and validation. PM, LC, LB, and RO: methodology and validation. EP: conceptualization, methodology, writing—original draft, writing—review and editing, and project administration. All authors made important contributions to this manuscript. All authors contributed to the article and approved the submitted version.
