# Supplementary material for: Macromineral requirements for maintenance and growth in male and female hair sheep
Source: Front Vet Sci. 2023 Mar 2;10:1032429. doi: 10.3389/fvets.2023.1032429 (PMC10017769; doi:10.3389/fvets.2023.1032429)
Supplement: Supplementary file 1 [file Table_1.docx]

Supplementary Material

Macromineral requirements for maintenance and growth in male and female hair sheep

Caio J.L. Herbster^1^, Matheus L.C. Abreu^2^, Antonio S. Brito Neto^1^, Marcilio S. Mendes^1^, Luciano P. da Silva^1^, Marcos I. Marcondes^3^, Pedro H. S. Mazza^4^, Luciano S. Cabral^2^, Leilson R. Bezerra^5^, Ronaldo L. Oliveira^4^, Elzania S. Pereira^1*^

*** Correspondence:** Elzania S. Pereira elzania@hotmail.com

**Supplementary TABLE 1.** Mineral body composition of reference animals.

| **Study** | **BW (kg)** | **EBW (kg)** | **Ca (g)** | **P (g)** | **K (g)** | **Mg (g)** | **Na (g)** |
| --- | --- | --- | --- | --- | --- | --- | --- |
| **Non-Castrated males** |  |  |  |  |  |  |  |
| Pereira et al. (15) | 13.57 | 9.23 | 97.30 | 70.72 | 20.47 | 3.48 | 24.42 |
| Silva et al.(16) | 12.71 | 12.23 | 159.68 | 97.92 | 9.28 | 3.49 | 5.90 |
| Pereira et al.(18) | 13.53 | 10.44 | 118.35 | 55.85 | 22.57 | 2.91 | 15.17 |
| Pereira et al. (17) | 15.00 | 10.00 | 141.58 | 103.04 | 24.02 | 7.76 | 18.99 |
| Pereira et al. (5) | 15.74 | 10.80 | 135.36 | 103.44 | 23.70 | 6.11 | 18.20 |
| **Castrated males** |  |  |  |  |  |  |  |
| Cabral et al. (14) | 15.37 | 12.12 | 112 | 54.00 | 12.12 | 3.64 | 9.69 |
| Silva et al. (16) | 11.69 | 11.26 | 162.14 | 94.15 | 6.40 | 3.47 | 5.31 |
| Pereira et al. (5) | 14.70 | 10.88 | 138.57 | 101.51 | 24.17 | 6.56 | 19.43 |
| Pereira et al.(17) | 15.03 | 10.30 | 110.43 | 86.15 | 23.04 | 6.05 | 17.13 |
| **Females** |  |  |  |  |  |  |  |
| Silva et al.(16) | 10.55 | 9.88 | 119.98 | 73.09 | 5.69 | 2.77 | 4.38 |
| Pereira et al. (5) | 15.60 | 11.10 | 170.33 | 103.23 | 20.80 | 7.00 | 18.85 |

*BW, Body weight; EBW, empty body weight*
